# Beneficial haemodynamic effect of indomethacin during endotoxin shock in anaesthetized pigsputative involvement of nitric oxide?

**DOI:** 10.1155/S0962935195000202

**Published:** 1995-03

**Authors:** T. Mózes, E. M. van Gelderen, E. J. Mylecharane, P. R. Saxena

**Affiliations:** 1 Department of Pharmacology Cardiovascular Research Institute ‘COEUR’, Faculty of Medicine and Health Sciences Erasmus University Rotterdam Postbox 1738 Rotterdam 3000 DR The Netherlands; 2 Department of Traumatology Semmelweis University of Medical Sciences Péterfy Hospital P.O. Box 76 Budapest 1441 Hungary; 3 Department of Pharmacology University of Sydney Australia

## Abstract

Endotoxin shock was induced in 31 anaesthetized pigs by infusion of 5 μg/kg of Escbeicbia coli endotoxin (LPS) over 60 min into the superior mesenteric artery. Fifteen of these pigs died within 30 min of the start of LPS infusion whereas the remaining 16 survived the experimental period of 2 h. In a group of nine pigs indomethacin (2 mg/kg, i.v.)was inected 20–25 rain after the start of LPS infusion at which time mean arterial blood pressure (MABP) had decreased below 40 mmHg indicating imminent death. Indomethacin immediately reversed the hypotension. In another group of five pigs, N^G^-nitro L-arginine-methyl ester (L-NAME, 1 and 3 mg/kg)was iniected 10 and 5 min, respectively, before the expected death without any beneficial effect on the hypotension. Three rain after the last dose of L-NAME, indomethacin (2 mg/kg, i.v.) was iniected. In three animals the hypotension was reserved by indomethacin, although this beneficial effect was delayed in comparison with the LP-Streated group not receiving L-NAME. Four pigs were pretreated with L-NAME, 3 mg/kg, i.v., 10 min prior to LPS infusion. All pretreated animals tended to die within 30 min of the start of the LPS infusion. Five rain before the expected death (20–25 rain after the start of LPS infusion) indomethacin (2 mg/kg) was inected. In three of these animals indomethacin reversed hypotenston and prevented death. Interestingly, this rise in the MABP developed very slowly. These results suggest that the beneficial effect of indomethacin in endotoxin shock might be related partially to interference with nitric oxide, which is not the only factor determining blood pressure levels during endotoxic shock.


Mediators of Inflammation 4, 117–123 (1995)

### Beneficial haemodynamic effect of indomethacin during endotoxin shock in anaesthetized pigs: putative involvement of nitric oxide?

T. Mózes,*[,CA] E. M. van Gelderen, E. J. Mylecharane† and P. R. Saxena

Department of Pharmacology, Cardiovascular Research Institute 'COEUR', Faculty of Medicine and Health Sciences, Erasmus University Rotterdam, Postbox 1738, 3000 DR Rotterdam, The Netherlands
Present addresses: *Department of Traumatology, Semmelweis University of Medical Sciences, Péterfy Hospital, P.O. Box 76, 1441 Budapest, Hungary. †Department of Pharmacology, University of Sydney, Australia.

[CA] Corresponding Author

**ENDOTOXIN shock was induced in 31 anaesthetized pigs by infusion of 5 μg/kg of *Escherichia coli* endotoxin (LPS) over 60 min into the superior mesenteric artery. Fifteen of these pigs died within 30 min of the start of LPS infusion whereas the remaining 16 survived the experimental period of 2 h. In a group of nine pigs indomethacin (2 mg/kg, i.v.) was injected 20–25 min after the start of LPS infusion at which time mean arterial blood pressure (MABP) had decreased below 40 mmHg indicating imminent death. Indomethacin immediately reversed the hypotension. In another group of five pigs, $N^G$-nitro-L-arginine-methyl ester (L-NAME, 1 and 3 mg/kg) was injected 10 and 5 min, respectively, before the expected death without any beneficial effect on the hypotension. Three min after the last dose of L-NAME, indomethacin (2 mg/kg, i.v.) was injected. In three animals the hypotension was reserved by indomethacin, although this beneficial effect was delayed in comparison with the LPS-treated group not receiving L-NAME. Four pigs were pretreated with L-NAME, 3 mg/kg, i.v., 10 min prior to LPS infusion. All pretreated animals tended to die within 30 min of the start of the LPS infusion. Five min before the expected death (20–25 min after the start of LPS infusion) indomethacin (2 mg/kg) was injected. In three of these animals indomethacin reversed hypotension and prevented death. Interestingly, this rise in the MABP developed very slowly. These results suggest that the beneficial effect of indomethacin in endotoxin shock might be related partially to interference with nitric oxide, which is not the only factor determining blood pressure levels during endotoxic shock.**

**Key words:** Endotoxin, Indomethacin, L-NAME, Nitric oxide, Pig, Shock

### Introduction

It is becoming increasingly clear that septic shock represents a generalized inflammatory reaction with the induction of a number of mediators similar to local inflammatory reactions.[1,2] Endotoxin and other bacterial products also induce the production of powerful mediators like eicosanoids, platelet-activating factor and cytokines.[3] Recently, nitric oxide (NO) has been suggested to be involved in the hypotension observed during endotoxin shock in dogs and rats.[4,5]

Non-steroid anti-inflammatory drugs (NSAIDs) represent a rational approach to break up the chain of mediators because of their ability to inhibit the cyclooxygenase enzyme, and this action may account for the clinical effectiveness of NSAIDs.[6] We have previously demonstrated that the administration of indomethacin during endotoxin shock prevented death and improved the shock state.[7] The beneficial effect on blood pressure was rapid in onset, reaching its maximum with 60 s, indicating a direct vasoconstrictor effect.[7] Since blood pressure was restored with 90 s of inhibition of NO synthesis during endotoxin shock in dogs,[4] one can assume some interrelationship between the effects of indomethacin and NO. The aim of the present investigation was to explore the existence of such a relationship.

### Materials and Methods

*General:* After an overnight fast, female Yorkshire × Landrace pigs (body weight, 20–24 kg; age, 12–15 weeks) were initially anaesthetized with i.m. injections of ketamine (20 mg/kg) and midazolam (0.25 mg/kg) as well as atropine (0.005 mg/kg). The anaesthesia was maintained throughout the experiment with i.v. sodium pentobarbitone (20 mg/kg bolus followed by 20 mg/kg/h infusion). After intubation the animals were ventilated with intermittent positive pressure (Bear 2E adult volume ventilator, Lameris, The Netherlands). Respiratory rate, tidal volume and oxygen to air ratio were adjusted to provide arterial blood gases within normal ranges at baseline: pH 7.35–7.45, $pO_2$ 90–150 mmHg and $pCO_2$ 35–45

©1995 Rapid Communications of Oxford Ltd mmHg. Arterial blood gases and pH were measured with a blood gas analyser (ABL-510, Radiometer, Copenhagen, Denmark). Body temperature, measured with a thermometer (Philips, HP 5311, Japan) attached to the liver, was maintained at around 38°C by using an electric blanket.

Catheters were placed via the femoral vessels into the aorta and pulmonary artery (Swan–Ganz 7F catheter). A midline laparotomy was performed and the superior mesenteric artery was exposed. After gently clearing the surrounding fat, nerves and connective tissues, a needle (0.5 mm external diameter) connected to a suitable polyethylene tube was inserted directly into the superior mesenteric artery for infusion of LPS or normal saline. The abdomen was then closed and a Ringer–lactate infusion at a rate of 4 ml/kg/h was started. The animals were allowed to remain undisturbed for at least 30 min to ensure haemodynamic and respiratory stability. Mean arterial blood pressure (MABP) and mean pulmonary arterial pressure (MPAP) were continuously monitored by electromanometers with Statham P23 dB strain gauges (Hato Rey, Puerto Rico). Cardiac output (CO) was determined intermittently by thermodilution (WTI Computer, Rotterdam, Holland); each measurement was performed in triplicate and their readings averaged.

*Experimental protocols*:

*Group I, Sham operated.* Five animals were prepared as described above except that instead of LPS (see Group II), normal saline was infused into the superior mesenteric artery.

*Group II, LPS induced shock.* Thirty-one pigs were infused with 5 μg/kg *Escherichia coli* endotoxin (LPS, O111 B4, Serva) into the superior mesenteric artery over a 60-min period and the animals were observed for an additional 60-min period.

*Group III, indomethacin revived.* In nine pigs, just after the mean arterial blood pressure had decreased below 40 mmHg at 20–25 min after the start of the LPS infusion, indomethacin (2 mg/kg i.v.) was injected.

*Group IV, L-NAME post-LPS treatment, indomethacin revived.* In five pigs, just after the mean arterial blood pressure had decreased below 40 mmHg at 20–25 min after starting the LPS infusion, L-NAME (1 and 3 mg/kg) was given 10 and 5 min, respectively, before the expected death. Three min after the last dose of L-NAME, indomethacin (2 mg/kg, i.v.) was injected.

*Group V, L-NAME pre-LPS treatment, indo- methacin received.* Four animals were pretreated with L-NAME, 3 mg/kg, 10 min prior to LPS infusion. All animals tended to die at or close to the critical 30 min after the start of the LPS infusion. Five min before the expected death indomethacin (2 mg/kg, i.v.) was injected.

*Statistical analysis:* All values are expressed as mean ± S.E.M. The data were evaluated by a non-parametric two-way analysis of variance (Friedman test) followed by a Wilcoxon test to isolate differences between measurements performed during the control period and at different times during and after LPS infusion. The Mann–Whitney *U* test was performed to evaluate the differences between the groups. A *p* value of 0.05 or less was considered statistically significant for all tests.

### Results

*Animal survival:* A total of 49 pigs received a LPS infusion for 60 min (Groups II, III, IV and V); Group II consisted of 31 animals which were treated with LPS only. In this group, 15 animals died at or close to 30 min after the start of LPS infusion (non-survivors). The remaining 16 animals survived the experimental period of 2 h (i.e. 60 min after termination of the LPS infusion) though in a state of shock (survivors). None of the animals receiving indomethacin (2 mg/kg), 20–25 min after starting LPS infusion (Group III), died during the observation period of 60 min. L-NAME (1 and 3 mg/kg), administered 10 and 5 min respectively, before the expected death, failed to revive the animals. Four pigs pretreated with L-NAME (3 mg/kg) tended to die at or close to 30 min after starting LPS infusion. In six of nine animals receiving L-NAME (pre- or post-LPS), indomethacin prevented death at or close to 30 min after starting LPS infusion. However, this beneficial effect was markedly delayed compared with the group treated with LPS, but not receiving L-NAME.

*Systemic haemodynamics:* As depicted in Fig. 1, no changes in systemic haemodynamics were observed in sham-operated animals. In non-surviving pigs, LPS infusion decreased MABP to 29 ± 2 mmHg whereas MPAP was increased to 45 ± 2 mmHg and CO fell to 0.93 ± 0.1 l/min, shortly before death (30 min after the start of LPS infusion). In surviving pigs treated with LPS infusion, MABP gradually decreased to 71 ± 4 mmHg at the end of 60 min observation period (60 min after the end of LPS infusion); MPAP first increased to peak values at 30 min before decreasing slightly but remaining above baseline values; CO first decreased rapidly 15–30 min after the start of the LPS infusion and then gradually to about 59% of the baseline value (Fig. 1A).

In animals revived with indomethacin (Group III), MABP increased dramatically immediately after indomethacin, to stabilize from 60 s onwards around the baseline value. MPAP was reduced but remained slightly elevated, whereas CO was transiently elevated and then decreased to a similar level as in untreated LPS-infused survivors (Fig. 1B). A typical

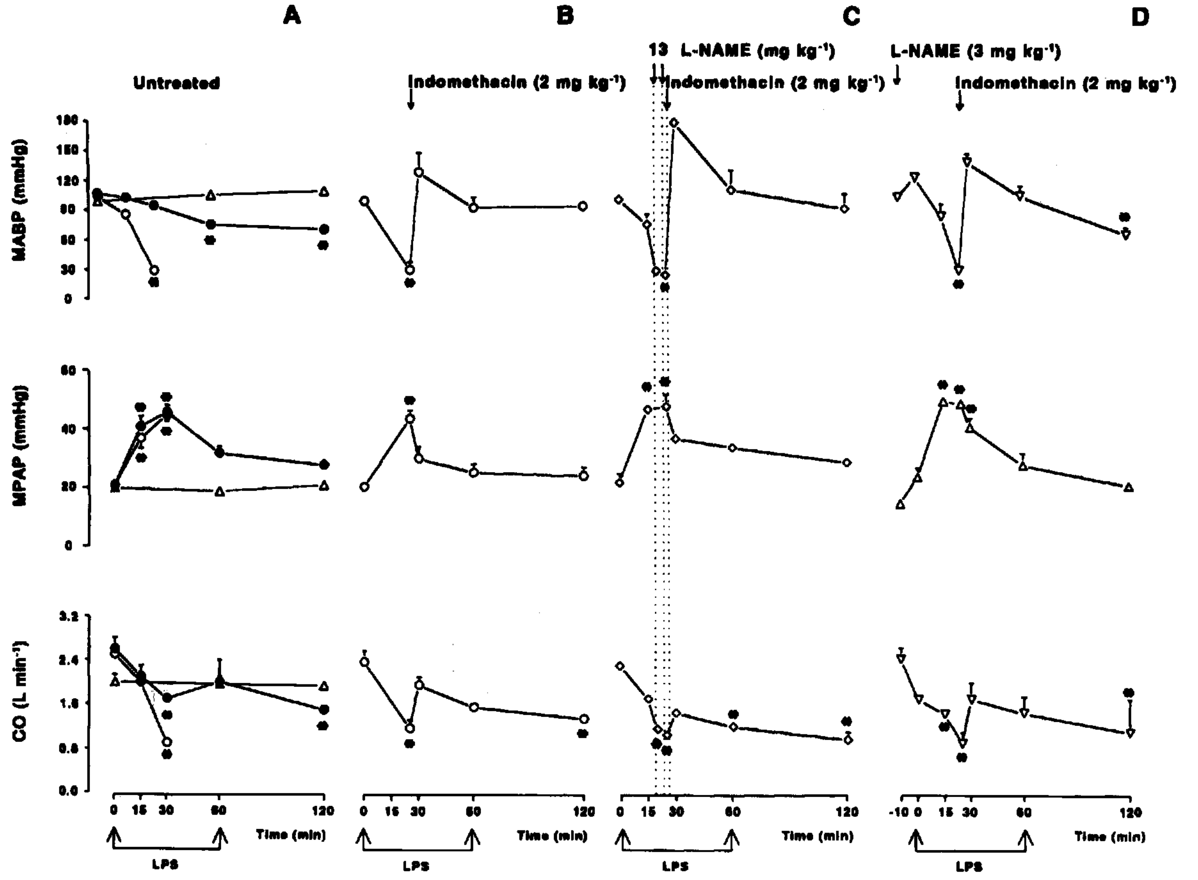

FIG. 1. The effect of saline or endotoxin (LPS, 5 μg/kg/h) infusion on systemic haemodynamics in untreated pigs (Panel A) and in pigs treated with indomethacin only (Panel B); in pigs treated with L-NAME + indomethacin during LPS infusion (Panel C) or in pigs treated with L-NAME before LPS infusion and with indomethacin during LPS infusion (Panel D). Panel A. (Δ), sham-operated saline-treated animals ($n = 5$); (○), non-surviving animals ($n = 15$) and (●), surviving animals ($n = 16$). Panel B, (○), non-survivors ($n = 9$) treated with indomethacin only. Panel C, (◇), non-survivors ($n = 5$) treated with L-NAME and indomethacin. Panel D, (∇), L-NAME pretreated animals ($n = 4$). Arrows indicate the time of administration of either LPS, L-NAME or indomethacin, as appropriate. Abbreviations: MABP, mean arterial blood pressure; CO, cardiac output; MPAP, mean pulmonary arterial pressure. Values are given as means ± S.E.M. *significantly different from baseline values ($p < 0.05$).

recording of the immediate reversal of MABP induced by indomethacin in an animal treated with LPS is shown in Fig. 2A.

In Group IV, five animals tended to die within 30 min after the start of LPS, and L-NAME (1 and 3 mg/kg), given 10 and 5 min, respectively, before the expected death, failed to prevent the drop in MABP (to 20 ± 2 mmHg) and the increase in MPAP (to 39 ± 4 mmHg) as well as the reduction in CO (to 1.0 ± 0.1 l/min). Three min after the last administration of L-NAME, indomethacin (2 mg/kg) was injected. This treatment was effective in restoring MABP in only three of five animals. Furthermore, in contrast to the immediate reversal of MABP observed in pigs treated with LPS but not receiving L-NAME, a delay (4 ± 1 min) was observed in the onset of the beneficial effect. MPAP declined after indomethacin treatment; CO was transiently elevated and then decreased to the same levels as in the untreated LPS-infused survivors (Fig. 1B and 2B).

In Group V, four animals pretreated with L-NAME (3 mg/kg) prior to LPS infusion, tended to die at or close to 30 min after the start of the LPS infusion. Indomethacin, given 5 min before the expected death, reversed the serious hypotension in three of four animals and prevented death. However, the delay between the indomethacin injection and the recovery of blood pressure was longer than in LPS-treated and/or L-NAME post-LPS treated groups (8 ± 0.6 min *vs* 1 ± 0.01 min and 4 ± 0.6 min, respectively). Furthermore, this recovery developed more slowly (in 13.5 ± 0.7 min instead of 60 s), a time that characterized the beneficial effect of indomethacin in pigs treated with LPS only. MPAP decreased after indomethacin treatment, whereas CO was transiently elevated and then decreased to a similar level as observed in the untreated LPS-infused survivors (Fig. 1C and 2C).

### Discussion

The main findings in the present study are: (1) LPS infusion into the superior mesenteric artery resulted in an almost even distribution of the experimental

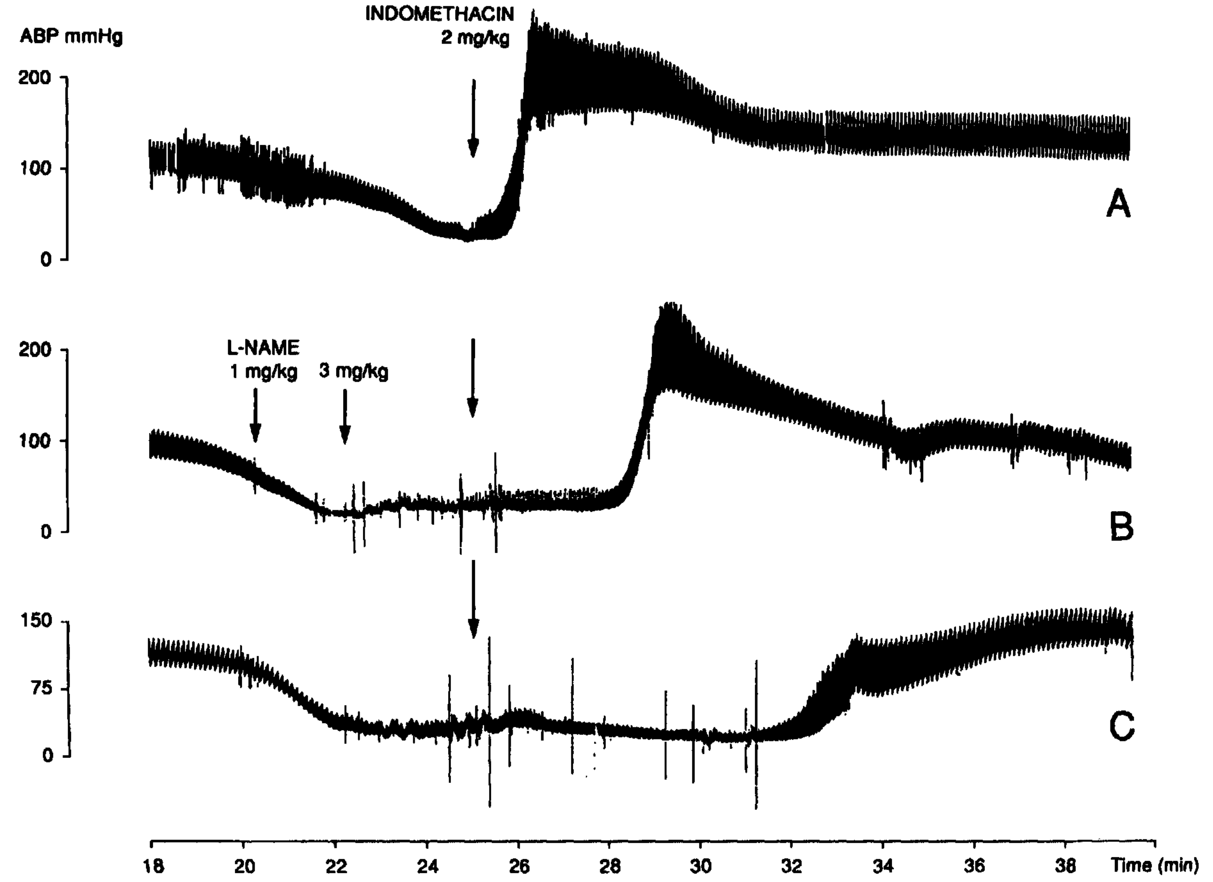

FIG. 2. Effect of i.v. treatment with indomethacin on arterial blood pressure (ABP) in anaesthetized pigs during endotoxin shock. Panel A, animals untreated with L-NAME (Group III); Panel B, following 1 and 3 mg/kg L-NAME given 5 and 10 min prior to indomethacin treatment (Group IV); and Panel C, after L-NAME pretreatment 5 min prior to the start of LPS infusion (Group V; time-point not shown). Arrows indicate the time of administration of either L-NAME or indomethacin, as appropriate. Time scale shows the time after the start of endotoxin infusion.

animals in two groups, non-survivors and survivors; (2) indomethacin administration, starting shortly before the presumed death, immediately reversed the hypotension and prevented death; (3) different doses of L-NAME, also given before the expected death, failed to restore blood pressure and prevent death; moreover, L-NAME delayed the onset of the beneficial effect of indomethacin treatment; and (4) L-NAME, administered 10 min prior to the LPS infusion markedly exacerbated the deleterious consequences of this infusion, resulting in a greater delay in the beneficial effect of indomethacin on haemodynamics than in animals not receiving L-NAME or in animals where L-NAME was administered after LPS treatment.

Continuous LPS infusion in pigs induced death in approximately half the animals and a prolonged state of shock was observed in the surviving animals (up to 1 h after termination of LPS infusion). The animals were treated with indomethacin at the time of the peak of LPS-induced hypotensive effect which was followed by death in untreated animals within 5–10 min. Indomethacin treatment was effective in preventing the imminent death and an immediate reversal of the hypotension was observed with a concomitant decrease in pulmonary artery pressure and an increase in cardiac output. This dose of indomethacin treatment effectively reduced the release of cyclooxygenase products suggesting that the beneficial effects of indomethacin treatment are due, at least in part, to the inhibition of prostanoid synthesis.[7] However, the effect of indomethacin on blood pressure was rapid in onset, reaching a maximum within 60 s, implying that the vasoconstriction may be independent of the inhibition of cyclooxygenase pathway.[7] Moreover, in non-survivors when indomethacin was effective, no changes were observed in the prostanoid release.[7] In a similar way, indomethacin restored the circulation during mesenteric ischaemia/reperfusion-induced shock in dogs; vasoconstriction developed in 30 s while the decrease in prostanoid concentration was observed later.[8] Although indirect vasoconstriction via suppression of local prostacyclin generation in a particular vascular bed, associated with a slower change in the circulatory 6-keto-$PGF_{1\alpha}$, cannot be entirely excluded, this does not seem very likely because both aspirin and ibuprofen, despite being potent cyclooxygenase inhibitors, are not capable of eliciting a rapid vasoconstrictor response.[9,10] The protective effect of ibuprofen against endotoxin shock develops with a delay of 45 min[10] and the improvement in the survival during endotoxin shock in dogs by aspirin occurs without any attenuation of the haemodynamic effects of LPS.[9]

Since blood pressure also returned to normal values within 90 s after inhibition of NO synthesis during endotoxin shock in dogs,[4] it is reasonable to assume some interrelationship between the effects of indomethacin and NO. However, in the present experiments L-NAME failed to reverse the hypotension suggesting that the use of inhibitors of both NO synthases ($Ca^{2+}$-dependent constitutive and $Ca^{2+}$-independent inducible) is not beneficial for treatment of shock in pigs. Similar effects following inhibition of NO synthases have been found in rabbits and rats as well as humans with endotoxin or septic shock indicating that inhibition of endogenous NO synthesis may be counter-productive.[5,11,12]

It may be argued that the inability of L-NAME to restore blood pressure in LPS-treated pigs is due to the dose of L-NAME employed in this study. However the highest dose (3 mg/kg) was effective in blocking NO synthesis as indicated by the reduced plasma $NO_2^-$ levels (Mózes *et al.*, unpublished observations). Furthermore, higher doses of L-NAME induced profound reductions in cardiac output limiting blood pressure increases in pigs[13] and failed to restore the haemodynamic changes induced by LPS infusion (Mózes *et al.*, unpublished observations).

Our findings are in accord with other studies, suggesting that some NO formation may be beneficial in endotoxin shock.[11,14,15] Indeed, NO production appears to be responsible for the maintenance of a low vascular tone within the pulmonary circulation.[15] Therefore, it seems likely that the expected rise in arterial blood pressure following blockade of NO synthesis is counteracted by the coincident fall in cardiac output due to pulmonary vasoconstriction in pigs.[13] The rapid increase in mean pulmonary arterial pressure and the consecutive decrease in cardiac output following LPS infusion were enhanced by any form of L-NAME treatment in the present experiments. These findings suggest that the vasodilator tone exerted by NO is important for maintaining lung perfusion in endotoxin shock, which view is further supported by the observation that the most pronounced NO induction following LPS can be found in the lung.[16] Interestingly, NO synthase activity in the aorta preceded the enzyme activity in the lung.[16] However, a negative inotropic effect of L-NAME, either directly on the myocardium or indirectly via coronary artery vasoconstriction contributing to the fall in cardiac output, cannot be excluded.[17,18] A recent study demonstrates that cytokines, including tumour necrosis factor (TNF), induce a negative inotropic effect on the heart mediated by cardiac NO production.[19] In addition, hypotension induced by TNF administration in dogs has been shown to be reversed by NO synthase inhibition[20] suggesting that the hypotension might be caused by an excessive NO production. Furthermore, in our endotoxic shock model, in non-survivors a marked increase was detected in plasma concentrations of TNF[7] and $NO_2^-$ (Mózes *et al.*, unpublished observations), but not platelet-activating factor or eicosanoids.[7] As hypotension following administration of either TNF or LPS occurred within 30 min, one may assume that NO might be produced by the constitutive NO synthase rather than the inducible isoform, since its induction requires 60–180 min.[16] Indeed, TNF has been reported to induce NO release within 5 min in the papillary muscle preparation.[19] These observations argue against an effect requiring gene transcription. Therefore, the negative inotropic effects of cytokines appear to result from enhanced activity of a constitutive NO synthase in myocardium.[19] Based on the above mentioned, it may be argued that the endotoxin-induced haemodynamic changes (hypotension and reduction in cardiac output) as well as early death in non-survivors were mediated in concert by TNF and NO. However, one should keep in mind that the constitutive NO synthase can produce only limited amounts of NO.[20] Thus, the observed haemodynamic changes can be evoked only partially by NO. Consequently, it seems that TNF may act directly as well as indirectly on the cardiovascular system in non-survivors.

Taking into account that NO seems to be responsible for the maintenance of the pulmonary circulation as well as the hypotension in endotoxin shock and that blockade of both isoforms of NO synthase may exacerbate the endotoxin shock due to the pulmonary insufficiency, the challenge is to restore blood pressure without suppression of the pulmonary circulation. The release of NO in the early and late phase of endotoxaemia is triggered by two distinct mechanisms; by activation of the constitutive and the inducible NO synthase isoforms, respectively.[16] It seems that with the use of non-selective NO synthase inhibitors, such as L-NAME and $N^G$-monomethyl-L-arginine (L-NMMA), the problem remains unsolved.[4,11,12] Selective inhibition of the inducible NO synthase has been advocated as a useful tool in the late phase of endotoxin shock.[11] In contrast, the enhanced formation of NO by the constitutive enzyme seems to account for the immediate hypotension in response to LPS[16] and a similar mechanism has been observed in haemorrhagic shock in anaesthetized rats.[22] Therefore, it is conceivable that the constitutive NO synthase should be blocked in the early phase of endotoxin shock. This view is supported by the observation that in the early phase of endotoxin shock the constitutive NO

synthase is more abundantly present in the aorta than in the lung.[16] Consequently, the use of an inhibitor of the constitutive enzyme may reverse the hypotension without disrupting the pulmonary homeostasis.

In the present study, indomethacin reversed hypotension, elevated cardiac output and prevented death. This beneficial effect on blood pressure was rapid in onset, reaching its maximum in 60 s, implying that vasoconstriction seen after indomethacin may be due to the inhibition of a rapidly degrading potent vasodilator like NO. However, the rapid release of a potent vasoconstrictor cannot be excluded. Nevertheless, our results demonstrate a long delay in the beneficial effect of indomethacin after the inhibition of NO synthesis; therefore, the release of a vasoconstrictor does not seem very likely. Furthermore, the observed delay in the beneficial effect of indomethacin seems to depend on the duration of inhibition of both NO synthases. Recently, we observed a significant reduction in plasma nitrite concentration following L-NAME (3 mg/kg) from 15 min to 120 min in pigs (Mózes *et al.*, unpublished observations). Since NO synthesis inhibition is more pronounced after 15 min, it is conceivable that in L-NAME pretreated animals the reversal of the hypotension takes longer (20–25 min after L-NAME) than in L-NAME post-LPS treated animals (3–8 min after L-NAME) or in the absence of L-NAME in LPS-treated animals. However, it is to be noted that the experiments were performed in pentobarbitone anaesthetized animals and it has been shown that this type of anaesthesia may delay the onset of pressor responses to L-NAME.[23] Hence it may be suggested that anaesthesia contributed in part to the more pronounced delay observed in L-NAME pretreated animals.

Apart from its cyclooxygenase inhibitory action indomethacin possesses other activities such as blocking calcium influx.[23,24] One possible explanation for the rapid restoration of blood pressure following indomethacin administration may be a selective inhibition of NO synthase. Since indomethacin inhibits $Ca^{2+}$ uptake,[24] the activity of the calcium-dependent NO synthase may be reduced. This view is supported by the observation that NO release is reduced by indomethacin-isolated rabbit hearts following reperfusion[25] and that nifedipine, a calcium channel antagonist, inhibits the induction of NO synthase by LPS.[26] These observations suggest that the rapid beneficial effect of indomethacin on haemodynamics in endotoxic shock may be mediated by some reduction in NO production or release. However, one should keep in mind that indomethacin demonstrated its beneficial effect on the circulation after a certain delay following L-NAME treatment suggesting that NO is not the only factor determining the blood pressure during endotoxic shock.

Finally, by increasing the intracellular cAMP level,[24] indomethacin may alter the balance between cAMP and cGMP. Because of their opposing effects on the circulation and platelet aggregation,[27] the balance between cAMP and cGMP might be more important than the change in their respective levels. Since NO is known to elevate cGMP levels, this explanation also suggests some interference between indomethacin and NO.

In summary, regardless of the precise mechanism, indomethacin administration during the early phase of LPS-induced shock reversed hypotension, decreased pulmonary pressure and improved cardiac output. It should be noted that the effect of indomethacin on haemodynamics preceded the effective blockade of cyclooxygenase enzyme.[7] Indomethacin was less effective after the inhibition of both NO synthases suggesting that the beneficial effect of indomethacin in endotoxin shock might be partly related to either selective inhibition of the ($Ca^{2+}$-dependent) NO synthase, to antagonism of NO itself or both. However, the observation that the beneficial effect of indomethacin was delayed after NO synthase inhibition and was less effective suggests that NO is not the only factor determining the hypotension during endotoxic shock.

### References

1. Vadas P, Hay JB. Involvement of phospholipase $A_2$ in the pathogenesis of the haemodynamic changes in endotoxin shock. *Can J Physiol Pharmacol* 1993; **61**: 561–566.
2. Dinarello CA. The proinflammatory cytokines interleukin 1 and tumour necrosis factor and the treatment of the septic shock syndrome. *J Infect Dis* 1991; **163**: 1177–1184.
3. Morrison DC, Ryan JL. Endotoxins and disease mechanisms. *Ann Rev Med* 1987; **38**: 417–432.
4. Kilbourn RG, Jubran A, Gross SS, Griffith OW, Levi R, Adams J, Lodato RF. Reversal of endotoxin-mediated shock by $N^G$-methyl-L-arginine, an inhibitor of nitric oxide synthesis. *Biochem Biophys Res Commun* 1990; **172**: 1132–1138.
5. Nava E, Palmer RMJ, Moncada S. Inhibition of nitric oxide synthesis in septic shock: how much is beneficial? *Lancet* 1991; **338**: 1555–1557.
6. Vane JR. Inhibition of prostaglandin synthesis as a mechanism of action for aspirin-like drugs. *Nature (New Biol)* 1971; **231**: 232–235.
7. Mózes T, Zijlstra FJ, Heiligers JPC, Tak CJAM, Ben-Efraim S, Bonta IL, Saxena PR. Sequential release of tumour necrosis factor, platelet activating factor and eicosanoids during endotoxin shock in anaesthetized pigs: protective effects of indomethacin. *Br J Pharmacol* 1991; **104**: 691–699.
8. Mózes T, Wieszt E, Zahajsky T. Role of $PGF_{2\alpha}$ in the superior mesenteric artery induced shock. *J Surg Res* 1989; **47**: 476–481.
9. Fletcher JR, Ramwell PW. Modification, by aspirin and indomethacin, of haemodynamic and prostaglandin releasing effects of *E. coli* endotoxin in the dog. *Br J Pharmacol* 1977; **61**: 175–181.
10. Balk RA, Jacobs RF, Tryka F, Walls RL, Bone RC. Low ibuprofen reverses the haemodynamic alterations of canine endotoxin shock. *Crit Care Med* 1988; **16**: 1128–1131.
11. Wright CE, Rees DD, Moncada S. Protective and pathological roles of nitric oxide in endotoxin shock. *Cardiovasc Res* 1992; **26**: 48–57.
12. Petros A, Lamb G, Leone A, Moncada S, Bennet D, Vallance P. Effects of a nitric oxide synthase inhibitor in humans with septic shock. *Cardiovasc Res* 1994; **28**: 34–39.
13. Van Gelderen EM, Den Boer MO, Saxena PR. $N^G$-nitro L-arginine methyl ester: systemic and pulmonary haemodynamics, tissue blood flow and arteriovenous shunting in the pig. *Naunyn Schmiedebergs Arch Pharmacol* 1993; **348**: 417–423.
14. Hutcheson IR, Whittle BJR, Boughton-Smith NK. Role of nitric oxide in maintaining vascular integrity in endotoxin-induced acute intestinal damage in the rat. *Br J Pharmacol* 1990; **101**: 815–820.
15. Weitzberg E, Rudehill A, Lundberg JM. Nitric oxide inhalation attenuates pulmonary hypertension and improves gas exchange in endotoxin shock. *Eur J Pharmacol* 1993; **233**: 85–94.
16. Szabó CS, Mitchell JA, Thiemermann G, Vane JR. Nitric oxide-mediated hyporeactivity to noradrenaline precedes the induction of nitric oxide synthase in endotoxin shock. *Br J Pharmacol* 1993; **108**: 786–792.

17. Gardiner SM, Compton AM, Kemp PA, Bennett T. Regional and cardiac haemodynamic effects of $N^G$-nitro-L-arginine methyl ester in conscious, Long Ewans rats. *Br J Pharmacol* 1990; **101**: 632–639.
18. Kelm M, Schroder J. Control of coronary vascular tone by nitric oxide. *Circ Res* 1990; **66**: 1561–1575.
19. Finkel MS, Oddis CV, Jacob TD, Watkins SC, Hattler BG, Simmons RL. Negative inotropic effects of cytokines on the heart mediated by nitric oxide. *Science* 1992; **257**: 387–389.
20. Kilbourn RG. Antagonism of tumour necrosis factor induced hypotension by $N^G$-monomethyl-L-arginine. In: Moncada S, Higgs EA, eds. *Nitric Oxide from L-Arginine: a Bioregulatory System*. Elsevier, 495–496.
21. Thiemermann C, Szabó CS, Mitchell JA, Vane JR. Vascular hyporeactivity to vasoconstrictor agents and haemodynamic decompensation in haemorrhagic shock is mediated by nitric oxide. *Proc Natl Acad Sci USA* 1993; **90**: 267–271.
22. Wang Y-X, Zhou T, Chua TC, Pang CCY. Effects of inhalation and intravenous anaesthetic agents on pressor response to $N^G$-nitro-L-arginine. *Eur J Pharmacol* 1991; **198**: 183–188.
23. Northover BJ. Effect of anti-inflammatory drugs on the binding of calcium to cellular membranes in various human and guinea-pig tissues. *Br J Pharmacol* 1973; **48**: 496–504.
24. Abramson S, Korchak H, Ludewig R, *et al*. Modes of action of aspirin-like drugs. *Proc Natl Acad Sci USA* 1985; **82**: 7227–7231.
25. Woditsch I, Schrör K. Prostacyclin rather than endogenous nitric oxide is a tissue protective factor in myocardial ischaemia. *Am J Physiol* 1992; **263**: H1390–H1396.
26. Szabó CS, Mitchell JA, Gross SS, Thiemermann C, Vane JR. Nifedipine inhibits the induction of nitric oxide synthase by bacterial lipopolysaccharide. *J Pharmacol Exp Ther* 1993; **265**: 674–680.
27. Moncada S, Palmer RMJ, Higgs EA. Nitric oxide; physiology, pathophysiology and pharmacology. *Pharmacol Rev* 1991; **43**: 109–142.

ACKNOWLEDGEMENTS. This work was funded by grants from the Hungarian Academy of Sciences (OTKA 1115), Erasmus University Foundation (Stichting Universiteitsfonds Rotterdam) and the Emil Starkenstein Foundation. The authors appreciate the excellent laboratory assistance of Mr J. P. C. Heiligers.

**Received 28 September 1994; accepted in revised form 8 December 1994**

## References

[PDF_00622] Abramson S., Korchak H., Ludewig R., Edelson H., Haines K., Levin R. I., Herman R., Rider L., Kimmel S., Weissmann G. (1985). Modes of action of aspirin-like drugs.. Proc Natl Acad Sci U S A.

[PDF_00583] Balk R. A., Jacobs R. F., Tryka A. F., Walls R. C., Bone R. C. (1988). Low dose ibuprofen reverses the hemodynamic alterations of canine endotoxin shock.. Crit Care Med.

[PDF_00562] Dinarello C. A. (1991). The proinflammatory cytokines interleukin-1 and tumor necrosis factor and treatment of the septic shock syndrome.. J Infect Dis.

[PDF_00610] Finkel M. S., Oddis C. V., Jacob T. D., Watkins S. C., Hattler B. G., Simmons R. L. (1992). Negative inotropic effects of cytokines on the heart mediated by nitric oxide.. Science.

[PDF_00580] Fletcher J. R., Ramwell P. W. (1977). Modification, by aspirin and indomethacin, of the haemodynamic and prostaglandin releasing effects of E. coli endotoxin in the dog.. Br J Pharmacol.

[PDF_00605] Gardiner S. M., Compton A. M., Kemp P. A., Bennett T. (1990). Regional and cardiac haemodynamic responses to glyceryl trinitrate, acetylcholine, bradykinin and endothelin-1 in conscious rats: effects of NG-nitro-L-arginine methyl ester.. Br J Pharmacol.

[PDF_00594] Hutcheson I. R., Whittle B. J., Boughton-Smith N. K. (1990). Role of nitric oxide in maintaining vascular integrity in endotoxin-induced acute intestinal damage in the rat.. Br J Pharmacol.

[PDF_00608] Kelm M., Schrader J. (1990). Control of coronary vascular tone by nitric oxide.. Circ Res.

[PDF_00567] Kilbourn R. G., Jubran A., Gross S. S., Griffith O. W., Levi R., Adams J., Lodato R. F. (1990). Reversal of endotoxin-mediated shock by NG-methyl-L-arginine, an inhibitor of nitric oxide synthesis.. Biochem Biophys Res Commun.

[PDF_00632] Moncada S., Palmer R. M., Higgs E. A. (1991). Nitric oxide: physiology, pathophysiology, and pharmacology.. Pharmacol Rev.

[PDF_00565] Morrison D. C., Ryan J. L. (1987). Endotoxins and disease mechanisms.. Annu Rev Med.

[PDF_00578] Mózes T., Wieszt E., Zahajszky T. (1989). Role of PGF2 alpha in the superior mesenteric artery-induced shock.. J Surg Res.

[PDF_00574] Mózes T., Zijlstra F. J., Heiligers J. P., Tak C. J., Ben-Efraim S., Bonta I. L., Saxena P. R. (1991). Sequential release of tumour necrosis factor, platelet activating factor and eicosanoids during endotoxin shock in anaesthetized pigs: protective effects of indomethacin.. Br J Pharmacol.

[PDF_00570] Nava E., Palmer R. M., Moncada S. (1991). Inhibition of nitric oxide synthesis in septic shock: how much is beneficial?. Lancet.

[PDF_00588] Petros A., Lamb G., Leone A., Moncada S., Bennett D., Vallance P. (1994). Effects of a nitric oxide synthase inhibitor in humans with septic shock.. Cardiovasc Res.

[PDF_00629] Szabó C., Mitchell J. A., Gross S. S., Thiemermann C., Vane J. R. (1993). Nifedipine inhibits the induction of nitric oxide synthase by bacterial lipopolysaccharide.. J Pharmacol Exp Ther.

[PDF_00600] Szabó C., Mitchell J. A., Thiemermann C., Vane J. R. (1993). Nitric oxide-mediated hyporeactivity to noradrenaline precedes the induction of nitric oxide synthase in endotoxin shock.. Br J Pharmacol.

[PDF_00616] Thiemermann C., Szabó C., Mitchell J. A., Vane J. R. (1993). Vascular hyporeactivity to vasoconstrictor agents and hemodynamic decompensation in hemorrhagic shock is mediated by nitric oxide.. Proc Natl Acad Sci U S A.

[PDF_00559] Vadas P., Hay J. B. (1983). Involvement of circulating phospholipase A2 in the pathogenesis of the hemodynamic changes in endotoxin shock.. Can J Physiol Pharmacol.

[PDF_00572] Vane J. R. (1971). Inhibition of prostaglandin synthesis as a mechanism of action for aspirin-like drugs.. Nat New Biol.

[PDF_00619] Wang Y. X., Zhou T., Chua T. C., Pang C. C. (1991). Effects of inhalation and intravenous anesthetic agents on pressor response to NG-nitro-L-arginine.. Eur J Pharmacol.

[PDF_00597] Weitzberg E., Rudehill A., Lundberg J. M. (1993). Nitric oxide inhalation attenuates pulmonary hypertension and improves gas exchange in endotoxin shock.. Eur J Pharmacol.

[PDF_00627] Woditsch I., Schrör K. (1992). Prostacyclin rather than endogenous nitric oxide is a tissue protective factor in myocardial ischemia.. Am J Physiol.

[PDF_00586] Wright C. E., Rees D. D., Moncada S. (1992). Protective and pathological roles of nitric oxide in endotoxin shock.. Cardiovasc Res.

[PDF_00591] van Gelderen E. M., Den Boer M. O., Saxena P. R. (1993). NG-nitro L-arginine methyl ester: systemic and pulmonary haemodynamics, tissue blood flow and arteriovenous shunting in the pig.. Naunyn Schmiedebergs Arch Pharmacol.

